# Mixed T cell lineage chimerism in acute leukemia/MDS using pre-emptive donor lymphocyte infusion strategy—Is it prognostic?—a single-center retrospective study

**DOI:** 10.1038/s41408-021-00519-y

**Published:** 2021-07-12

**Authors:** Vipul Sheth, Victoria Potter, Hugues de Lavallade, Shreyans Gandhi, Austin Kulasekararaj, Pramila Krishnamurthy, Varun Mehra, Francesco Dazzi, Ghulam Mufti, Antonio Pagliuca, Donal Mclornan, Kavita Raj

**Affiliations:** 1 Department of Haematological malignancies and stem cell transplant, Kings College hospital NHS trust, London, UK; 2 Department of Haematological malignancies, Guys and St Thomas hospital NHS trust, London, UK; 3 Present Address: Clinical Research Division, Program in Immunology, Fred Hutchinson Cancer Research Center, Seattle, WA USA

**Keywords:** Myelodysplastic syndrome, Leukaemia

## Abstract

Pre-emptive DLI (pDLI) is an effective strategy in lowering the risk of relapse without significantly increasing the risk of graft-versus-host disease (GVHD) in the case of T cell lineage mixed chimerism (MC) post allogeneic transplant in hematological malignancies. Many patients, however, fail to receive timely pDLI and have dismal outcomes, which are not taken into consideration. We compared long-term outcomes of 106 patients having T cell MC after day 60 and undergoing allogeneic stem cell allograft for acute leukemia from an unrelated donor (UD), with 111 patients having complete chimerism (CC). Fifty-three (56%) patients received prophylactic pDLI. Thirty-six patients (67%) had a response (RR), 17 (33%) had no response (NR), and fifty-two (54%) did not receive any pDLI (ND). OS was better in MC group as compared to CC (54% vs 43%, *p* = 0.04), mainly due to reduction in NRM (14% vs 25%, *p* = 0.05), and all grade acute and chronic GVHD. Within the MC group, response to pDLI was the only significant factor predicting OS, DFS, and relapses with NR and ND having unfavorable outcomes as compared to RR (*p* = 0.001). T cell MC in patients undergoing UD allografts with alemtuzumab is no longer an adverse prognostic factor, as compared to patients having CC, after timely implementation of pDLI.

## Introduction

Allogeneic hematopoietic stem cell transplantation from unrelated donors (UD) using peripheral stem cell grafts (PBSC) is effective in hematological malignancies but has been associated with relatively high rates of both acute and chronic graft-versus-host disease (GVHD) resulting in inferior survival as compared to matched donors (MSD) [1, 2].

T-cell depletion, especially alemtuzumab, reduces the risk of GVHD following allogeneic hematopoietic stem cell transplantation (HSCT) [3], but has been associated with an increased frequency of mixed T cell chimerism (MC) [4, 5]. Although stable MC (T cell subset), because of bilateral tolerance, has been associated with less acute GVHD [4–6], progressive loss of donor chimerism is associated with graft rejection [7–9] and disease relapse [6, 10–12]. The incidence of relapses and rejection further varies according to the intensities of the conditioning regimen [13]. In patients with persistent MC, donor lymphocyte infusion (pDLI) has been utilized to pre-emptively convert patients to full donor and prevent relapse [8, 14–17]. Majority of the studies demonstrating patients receiving pDLI and achieving outcomes similar to patients having complete chimerism (CC-mainly responders) were in pediatric settings, while using antithymocyte globulin [18, 19] and chimerism in CD34/33 fraction. In adults and alemtuzumab-based settings [20], the effectiveness of this strategy was demonstrated in achieving complete donor chimerism, optimal tolerance and outcomes [21, 22] and better efficacy as compared to therapeutic DLI [23], in patients receiving reduced-intensity conditioning regimens. However, realistically many patients with MC actually fail to receive immunotherapy, and they continue to have poor prognosis [22], which was not taken into consideration

We thus wanted to compare MC as a group (including patients unable to receive pDLI) to patients having CC and see if it still remains a prognostic factor after the early introduction of pDLI.

## Methods and materials

We retrospectively analyzed 224 patients who received first allo-HCT for acute myeloid leukemia (AML), myelodysplastic syndrome (MDS), myeloproliferative neoplasm (MPN), and acute lymphoblastic leukemia (ALL) transplanted from UD allograft (matched or mismatched) between the years 2007–2015. Out of 224 patients, 106 patients (47%) had T cell MC beyond day 60 of transplantation with complete lineage-specific chimerism (CD15/CD33/C19) and were compared to 111 patients having complete chimerism (CC) on day 60. Seven patients who could not be evaluated for day 60 chimerism were excluded from the analysis. All patients received in vivo T cell depletion with alemtuzumab. Patients having at least grade 2 GVHD prior to DLI were excluded from this strategy. The primary objective of this study was to analyze the long-term outcomes of patients with MC and whether pre-emptive DLI negates the adverse prognosis in terms of overall survival (OS). This study was reviewed and approved by the Institutional Review Boards of Kings College Hospital, and all patients were consented to as per the declaration of Helsinki.

### Conditioning regimen and GVHD prophylaxis

The majority of patients (218/224, 97%) received peripheral blood stem cell (PBSC) grafts. Seventy-four 74/224 (33%)—[39/108 (37%) in the MC group, 52/115 (43%) in the CC group, *p* = 0.30] patients received 60 mg of alemtuzumab (given days -6 through -4), and 150/224 (67%), [69/108 (63%) patients MC group, 63/115 (57%) in the CC group] patients received 100 mg of alemtuzumab (given days-8 through -4). Alemtuzumab was given on days-3 to -1 for Bu/Cy and Cy/TBI regimens. Changes in dosing over time reflect systematic changes in institutional practice. Additional GVHD prophylaxis included a calcineurin inhibitor and methotrexate/mycophenolate mofetil (MMF) for ablative conditioning and a calcineurin inhibitor for reduced-intensity conditioning. Conditioning regimen consisted of fludarabine and high-dose busulfan (16 mg/kg orally or 12.8 mg/kg i.v.) (FB4) or cyclophosphamide (120 mg/kg) and total body irradiation (1200 cGy) (Cy/TBI) or busulfan (16 mg/kg orally, or 12.8 mg/kg i.v.) and cyclophosphamide (120 mg/kg) (Bu/Cy) for patients receiving ablative conditioning, and fludarabine and busulfan (6.4 mg/kg i.v.) (FB2), or fludarabine and melphalan (140 mg/m^2^) (FM) for patients receiving reduced-intensity conditioning (RIC). Pre-transplant disease risk was represented using the Disease Risk Index [24]. Acute GVHD was assessed using modified Glucksberg criteria, and chronic GVHD was classified as limited or extensive according to NIH criteria [25, 26].

### Endpoints

OS was calculated from the time of transplant to the last follow-up date, Disease-free survival (DFS) was calculated as the time from transplantation and the earliest occurrence of any event relapse or death. Cytogenetic abnormality was classified according to the 2010 European Leukemia Net cytogenetic classification system [27]. The Chimerism was monitored using PCR amplification of a polymorphic STR region [28] every month for up to 90 days, and then every 3 months up to 2 years and then at 6-month intervals. Chimerism was separately analyzed for T cell subsets. The Indications for pDLI were falling T cell chimerism <50%. MC was defined as donor status less than 98% at day +60 (all data between day 50 to day 70). The first dose of pDLI was initiated at 5 × 10^5^/kg for UD and 1 × 10^6^/kg for siblings, and subsequent cycles were repeated at an interval of 6–8 weeks with escalating doses by half log each time, provided there was no evidence of active GVHD. The intention was to start pDLI by day 100. At least a 10% increase in chimerism, sustained at least for 2 months after the first pDLI was considered significant for a response (RR)

### Statistical methods

Categorical variables were summarized as frequency counts and compared using chi-squared tests, and continuous variables were summarized as medians and compared using Mann–Whitney U-tests. OS and DFS were estimated by the Kaplan–Meier method and compared using the log-rank test. Cumulative incidences of relapse, non-relapse mortality (NRM), and GVHD were compared using the Finney and Grey model (25) and were considered as competing risks for each other (26). The univariate analysis included the following factors: conditioning intensity, type of donor, disease risk index, age, time to initiation of pDLI, chimerism prior to pDLI, and cell dose. Variables significant at the univariate level (*p* = or < 0.1), or variables which could affect final outcomes were then entered into a multivariate cox proportional hazard model and logistic regression. Statistical analysis was conducted using SPSS 24.0 software and R 3.4.3, and a p-value of 0.05 was used to indicate statistical significance, and all tests were two sided.

## Results

### Demographic profile

All patients treated between 2007 and 2015 were included in the analysis. Sixty-six patients (63%) patients having MC were age above 52 years. Within the patients having MC, 27 patients (22%) had mismatched 9/10 grafts, 83 (78%) patients had intermediate disease risk index, 42 (40%) patients had a myeloablative regimen, and 99 (93%) patients had myeloid disorder (48% MDS). The median follow-up period was 33 months (range, 0.6–150 months) for both the groups, (Table 1). The demographic distribution was similar among patients having CC.

**Table 1:** Demographic characteristics of our study cohort.

Demographic profile	CC group *N* = 111	MC group *N* = 106	*P*-value
*Age (years)*			
**<**52	36 (33%)	40 (37%)	0.32
>52	75 (67%)	66 (63%)	
*Matching*			
9/10 (5 patients HLA DQ MM)	26 (25%)	27 (23%)	0.57
10/10	85 (75%)	79 (77%)	
*Disease risk index*			
Intermediate	81 (72%)	83 (78%)	0.34
High risk	30 (28%)	23 (22%)	
*Disease type*			
Myeloid	104 (93%)	99 (93%)	0.65
Lymphoid	7 (7%)	7 (7%)	
*Stem cell source*			
Peripheral blood	109 (98%)	104 (97%)	0.30
Bone marrow	2 (2%)	2 (3%)	
*Conditioning regimen*			
Reduced intensity	58 (52%)	64 (60%)	0.25
Myeloablative	53 (48%)	42 (40%)	

*pDLI* pre-emptive donor lymphocyte infusion, *MC* mixed chimerism, *CC* complete chimerism, *MM* mismatch.

### Donor lymphocyte infusion, chimerism, and response

Fifty-three (56%) patients received pDLI. The median dose of pDLI was 1 × 10^6^/kg (5 × 10^5^/kg–1 × 10^7^/kg) and the median time to pDLI was 5 months from transplantation (3.5–30 months). Patients received a median of 2 cycles of pDLI. The median CD3 at day 60 was 52%, with 37 (70%) of patients having CD3 chimerism <54%, (Supplementary Fig. 1). Out of 53 patients, 36 patients (67%) had a response (RR), and 17 (33%) patients had no response (NR). Fifty-two patients (54%) did not receive any DLI (ND). The main reason for patients unable to receive pDLI was either NRM (65% patients) or GVHD (unresolved grade 2 or higher grade GVHD, 28 %). Among 36 responders, the median time to best response was 7 months with the longest interval of the best response being around 100 months. Thirty-five patients among RR (98%) continued to maintain response beyond two months of starting pDLI and only one patient relapsed 5 months post-DLI and succumbed to progressive disease. None of the variables could significantly predict RR/NR.

### Overall survival and disease-free survival, (OS, DFS) and relapses

**Fig. 1:**
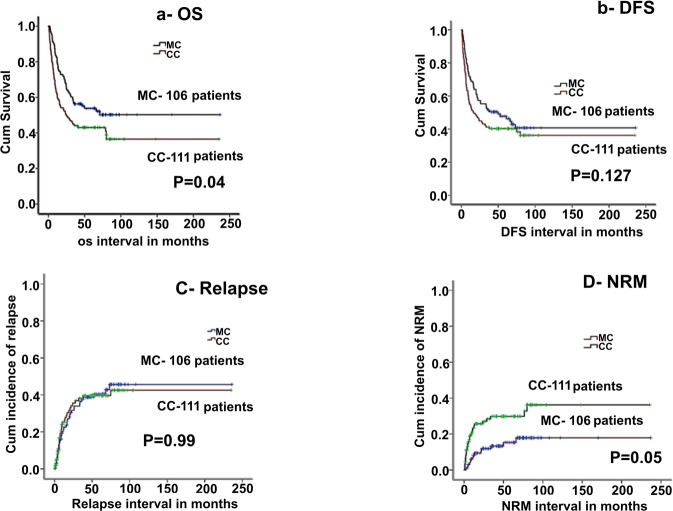
Comparison of outcomes between MC and CC. **a**, **b** The 5-year OS and DFS (CC-complete chimerism, MC mixed chimerism). **c**, **d** Relapses and NRM (CC complete chimerism, MC mixed chimerism).

**Fig. 2:**
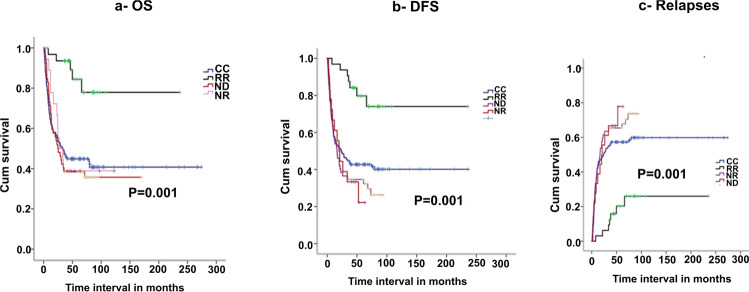
Comparison of outcomes between CC, RR, NR, and ND. **a**, **b** The 5-year DFS and OS (CC complete chimerism, RR responders, NR non-responder, ND No DLI). **c** Relapses (CC complete chimerism, RR responders, NR non-responder, ND No DLI).

5-year OS was worse in the CC group as compared to MC (43% vs 54%, *p* = 0.04). Two-year relapse and DFS between the groups were nearly similar (32% vs 38%, *p* = 0.99 and 38.5% vs 45%, *p* = 0.12, for CC and MC, respectively), (Fig. 1a–c). In multivariate analysis CC (ref MC, *p* = 0.02, HR: 1.53, CI: 1.0–2.2), older age, and mismatched transplant were independently predictive of the inferior OS, (Table 2). Among patients with MC, NR and ND patients did significantly worse as compared to RR in terms of DFS, OS, and relapse (*p* = 0.0001, HR = 5.45, 95% CI: 2.4–12, *p* = 0.001, HR-5.95, CI: 2.3–15, *p* = 0.0001, HR 9.45, CI: 2.8–30, respectively), (Fig. 2a–c, Table 3 and supplementary table 1).

**Table 2:** Cox regression-factors affecting OS and NRM in all the patients (MC and CC).

*N* = 217 patients	OS			NRM		
	HR (UV/MV)	CI (UV/MV)	P (UV/MV)	HR (UV/MV)	CI (UV/MV)	P (UV/MV)
Conditioning regimen (ref RIC)	1.2	0.8–1.83	0.21	1.7/1.9	0.9–3.1/1.07–3.5	0.07/0.06
Disease risk (DRI, ref low risk)	1.3	0.8–1.9	0.24	1.2	0.6–2.3	0.60
Age (ref < 52)	1.89/1.7	1.3–2.9/1.1–2.6	0.001/0.013	2.4 /2.7	1.3–5.2/1.3–5.7	0.03/0.04
Matching (ref 9/10)	0.60/0.65	0.4–0.8/0.43–0.9	0.02/0.019	0.65	0.3–1.2	0.20
MC vs CC (ref MC)	1.53/1.5	1.0–2.2/1.05–2.2	0.04/0.02	1.9/1.9	1.3–3.5/1.0–3.2	0.05/0.02

*OS* overall survival, *DFS* disease-free survival, *NRM* non-relapse mortality, *MA* myeloablative regimen, *RIC* reduced-intensity conditioning, *MC* mixed chimerism, *CC* complete chimerism, *pDLI* pre-emptive donor lymphocyte infusion, *NA* not applicable.

**Table 3:** Cox regression factors affecting OS/DFS/relapse in patients with MC.

*N* = 106 patients	OS(UV/MV)			DFS(UV/MV)			Relapse (UV/MV)		
	HR (UV/MV)	CI (UV/MV)	P (UV/MV)	HR (UV/MV)	CI UV/MV)	P (UV/MV)	HR (UV/MV)	CI (UV/MV)	P (UV/MV)
Conditioning regimen (ref RIC)	0.93	0.7–1.2	0.66	1.01	0.7–1.2	0.88	0.95	0.6–1.3	0.78
Disease risk (DRI, ref high risk)	1.1	0.5–2.3	0.65	1.17	0.6–2.2	0.61	1.12	0.5–2.4	0.76
Age (ref > 52)	1.34	0.7–2.3	0.34	1.12	0.5–1.9	0.58	1.11	0.6–2.1	0.73
Presence of cGVHD (ref yes)	1.08	0.5–2.0	0.79	0.83	0.4–1.4	0.53	0.76	0.3–1.5	0.45
Matching (ref 9/10)	0.64	0.3–1.2	0.17	0.75	0.4–1.3	0.31	0.73	0.3–1.5	0.39
CD 3 day 60 (ref < 50)	1.24	0.6–2.2	0.55	1.23	0.6–2.1	0.49	0.90	0.4–1.8	0.78
Response to pDLI (No response vs response)	5.95/5.9	2.3–15/2.3–15	0.001/ 0.001	5.45/5.4	2.4–12/2.4–12	0.0001/ 0.0001	9.45/9.4	2.8–30/2.8–30	0.0001/ 0.0001
Presence of severe cGVHD (ref yes)	1.42	0.6–3.5	0.37	1.09	0.4–2.5	0.82	0.78	0.2–2.5	0.68

*OS* overall survival, *DFS* disease-free survival, *GVHD* graft vs host disease, *BM* bone marrow, *NRM* non-relapse mortality, *MA* myeloablative regimen, *RIC* reduced-intensity conditioning, *MC* mixed chimerism, *pDLI* pre-emptive donor lymphocyte infusion.

### Non-relapse mortality (2 years, NRM)

Two-year non-relapse treatment-related mortality (NRM) was significantly higher in the CC group (mainly related to GVHD) as compared to MC (25% vs 14%, *p* = 0.05). After MV analysis, CC (ref MC, *p* = 0.02, HR: 2.44, CI: 1.3–5.2), and older age were independent variables predicting higher NRM (Fig. 1d and Table 2). Within the MC group, infections (bacterial, viral, or fungal) and NRM were comparable between pDLI (RR/NR), and ND patients, (*p* = 0.37). There was 70% reactivation of CMV reactivation, without any significant CMV disease in patients who received pDLI, and there was no fungal death within 100 days of pDLI. DLI was well tolerated and with an incidence of only 6% overall NRM within the first 100 days among patients who had a response to pDLI. Among patients who received pDLI, 9 patients died of progression of the disease (8 patients having NR and 1 patient having RR), 1 patient died each of infection, severe acute GVHD, and secondary malignancy (all having RR). Among patients who did not receive pDLI primary cause of mortality was disease progression (85%). Only 3 patients receiving pDLI had long-term aplasia and they eventually relapsed (disease-related).

### GVHD

**Fig. 3:**
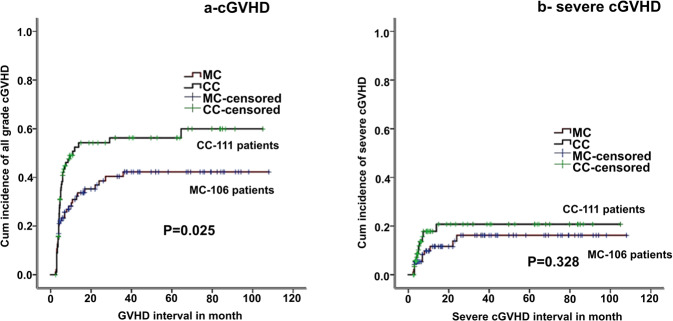
Comparison of cGVHD between CC and MC. **a** Cumulative incidence of all grade cGVHD (CC complete chimerism, MC mixed chimerism). **b** Cumulative incidence severe grade cGVHD (CC complete chimerism, MC mixed chimerism).

**Fig. 4:**
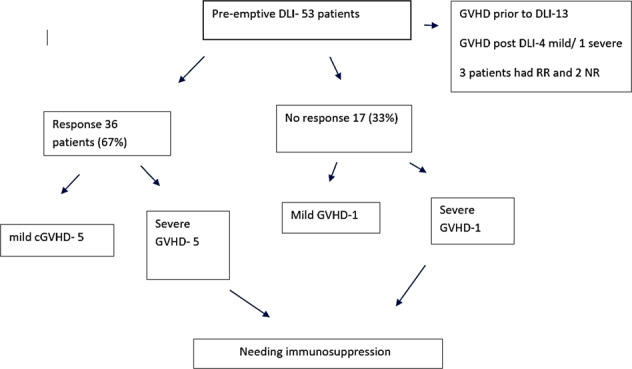
Graphical representation of patients who got GVHD after pDLI.

All grade acute GVHD (I–IV) was significantly lower in the MC group (60% vs 38%, *p* < 0.001), however, acute severe GVHD (grade III and IV) was similar between patients having CC and MC (11% vs 13%, *p* = 0.83). Incidence of all grade cGVHD was significantly more in the CC group than MC (48% vs 37%, *p* = 0.05), although, severe extensive cGVHD was similar between the two (14% vs 11%, *p* = 0.32 for CC and MC, respectively), (Fig. 3a, b). The median time to develop chronic severe GVHD was 6 months (range, 3–64 months) in the MC group. Within the MC group, 14 (12%) patients had GVHD prior to initiation of pDLI (completely resolved at the time of initiation of pDLI, 5 experienced recurrence after pDLI). The two-year incidence of severe GVHD was 12% and grade 1–2 GVHD was 32%, respectively (among patients who received pDLI), and was comparable to ND (Table 3, Fig. 4). The median time to onset of GVHD in patients receiving pDLI was 4 months (3–11 months). Six patients (38%) needed some immunosuppression after pDLI due to GVHD. The presence/absence of GVHD did not alter outcomes (DFS/OS) for patients having MC or among patients who received pDLI (supplementary table 1). However, patients achieving RR without GVHD had significantly better outcomes than patients with MC having NR or RR with GVHD [22].

## Discussion

T-cell depletion in UD grafts, especially alemtuzumab is associated with an increased frequency of mixed chimerism (MC) and relapses [4, 7, 11]. Pre-emptive DLI has been an effective strategy to prevent impending relapses without experiencing adverse graft vs host disease or infection in children and adults [15] However, studies have focused on comparing pDLI with patients having complete chimerism, not taking into account patients who fail to receive timely pDLI [15, 17] We retrospectively analyzed a large single-center dataset, of patients having mixed T cell chimerism (day 60) post allogeneic stem cell transplant in alemtuzumab-based setting, and compared their outcomes to patients having CC.

Overall, our results showed lower NRM associated with the MC group as compared to CC patients (mainly due to a reduction in overall GVHD). Despite relatively older patients (median age almost 60 years) and poor-disease risk index (61% high and intermediate risk), the non-relapse mortality was low (6% and 14% at 100 days and 1 year, respectively) among patients who received pDLI, and it was also associated with low rates of hospitalization as well as infections. Although CMV reactivation was commonly encountered, there were no cases of CMV disease, and no fungal infections or related deaths were seen in the first 100 days.

There was a significantly low incidence of all grades acute and chronic GVHD among patients who had MC as compared to patients having CC, though severe grade GVHD was comparable. The incidence of severe GVHD in patients receiving pDLI was comparable with that reported by Solomon et al. (12%) [21], wherein 36 patients, receiving alemtuzumab-based T cell depletion, received pDLI without withdrawal of immune suppression. It was lower than that reported by Bar et al. (28%) [29], where 35 patients prospectively received pDLI with pentostatin. The presence or absence of GVHD did not affect overall outcomes, similar to other groups. Although, severe GVHD was comparable between the two groups—probably the delay in onset of severe GVHD within the MC group might make it more amenable to treatment response and less aggressive as compared to early onset severe GVHD

OS was better in the MC group as compared to CC, especially among patients who received pDLI, and responded to the same (mainly due to reduction in NRM attributed to early GVHD within 100 days in the CC group). However, outcomes among patients within the MC group having ND and NR were particularly dismal. OS, DFS, and relapse, in our study, rates were significantly better for patients with MC as compared to other historical groups wherein pDLI was not implemented [4, 6, 12]. Within the MC group, the OS, DFS, and relapse rates of patients receiving pDLI or ND, in our cohort, was similar to that shown by Retrigger et al., (DFS: 66% for MC receiving pDLI, as compared to nearly 0% for patients not receiving DLI, *p* = 0.0009—among pediatric population using CD34/33 as chimerism fraction) [15], the study by Bar et al. (DFS: 45% for RR group vs 20% for NR) [21], and Solomon et al. (OS: 48% among 36 patients receiving pDLI) [29]. Also, we believe, that as outcomes in NR continues to remain very dismal, every attempt should be made in implementing additional pre-emptive novel agents (azacytidine/pentostatin) in the NR group. As some patients relapsing with leukemic blasts developed aGVHD despite the detection of MC or were critically ill, this recommended pre-emptive immunotherapy approach could not be offered to all MC patients. This is a drawback in most of the studies, and it would be worthwhile exploring other early immune-modulatory drugs (azacytidine) to further improve outcomes among patients having MC.

In univariate and multivariate analysis, the only factor predicting outcomes in patients with MC was the implementation of pDLI and the associated response, with the best outcomes among patients having a favorable response. The low CD3 percentage at day 60 of transplantation was not associated with response or overall outcomes, in contradiction to another study [16], wherein patients with high-risk disease had received only non-myeloablative conditioning. In a study by Bar et al. [29], where they used reduced-intensity conditioning along with pDLI with pentostatin, no such relation was found between CD3 and response. There was also no correlation in our study between the interval to initiation of pDLI and response or overall outcomes. Solomon et al. [21], in their study, showed a distinct improvement in outcomes in patients receiving pDLI within the first 100 days of transplantation. However, the subgroups were very small to have any definitive conclusions.

The retrospective nature of the study and a small subgroup of patients receiving pDLI (53 patients) among patients with MC, remains a potential drawback of this study. Furthermore, many patients in the MC group could not receive DLI due to the reasons mentioned above potentially biasing the results. Also, seven patients died prior to day 60 evaluation and were excluded from analysis, and although the number is very small it could still lead to a potential selection bias.

In conclusion, pre-emptive DLI (patients with T cell mixed chimerism after day 60) seems to be a successful and well-tolerated strategy in both the RIC and myeloablative setting to overcome adverse prognosis associated with MC, in adult patients with acute leukemia and undergoing unrelated donor transplant with alemtuzumab induced T cell depletion. Response to pDLI correlates strongly with final outcomes.

## Supplementary information

Supplementary table 1

Supplementary figure 1

BCJ-checklist
