# Supplementary material for: Mixed T cell lineage chimerism in acute leukemia/MDS using pre-emptive donor lymphocyte infusion strategy—Is it prognostic?—a single-center retrospective study
Source: Blood Cancer J. 2021 Jul 12;11(7):128. doi: 10.1038/s41408-021-00519-y (PMC8275738; doi:10.1038/s41408-021-00519-y)
Supplement: Supplementary file 1 — Supplementary table 1 [file 41408_2021_519_MOESM1_ESM.docx]

| N=53 patients | DFS (UV/MV) | | | OS (UV/MV) | | |
| --- | --- | --- | --- | --- | --- | --- |
|  | P | HR | CI | P | HR | CI |
| Disease risk (ref high risk) | 0.45 | 0.57 | 0.1-2.4 | 0.32 | 0.36 | 0.04-2.7 |
| Age (ref >52 years) | 0.95 | 0.97 | 0.3-2.4 | 0.72 | 1.21 | 0.4-3.5 |
| Matching (ref 9/10) | 0.34 | 0.63 | 0.24-1.6 | 0.07 | 0.39 | 0.1-1.1 |
| Conditioning (ref RIC) | 0.28 | 0.76 | 0.4-1.24 | 0.21 | 0.69 | 0.3-1.2 |
| pDLI interval within 5 months (ref >5 months) | 0.11 | 0.46 | 0.1-1.2 | 0.17 | 0.47 | 0.1-1.3 |
| CD3 day 60 >50 (ref <50) | 0.37 | 0.51 | 0.1-2.2 | 0.29 | 0.33 | 0.04-2.6 |
| Response to pDLI (ref response) | 0.0001/0.0001 | 7.04 | 2.6-18.8 | 0.001/0.001 | 6.21 | 2.1-18.2 |
| GVHD any grade, acute or chronic (ref yes) | 0.48 | 1.43 | 0.5-3.6 | 0.17 | 2.17 | 0.7-6.6 |

Table 1 (supplementary table), Cox regression- Factors affecting DFS and OS in patients receiving pDLI for MC

OS- overall survival, DFS- disease free survival, ALC – absolute lymphocyte counts of recipient, RIC- reduced intensity conditioning, pDLI-pre-emptive donor lymphocyte infusion, MC- mixed chimerism, GVHD- graft vs host disease
