# Supplementary figures and images for: Mixed T cell lineage chimerism in acute leukemia/MDS using pre-emptive donor lymphocyte infusion strategy—Is it prognostic?—a single-center retrospective study
Source: Blood Cancer J. 2021 Jul 12;11(7):128. doi: 10.1038/s41408-021-00519-y (PMC8275738; doi:10.1038/s41408-021-00519-y)

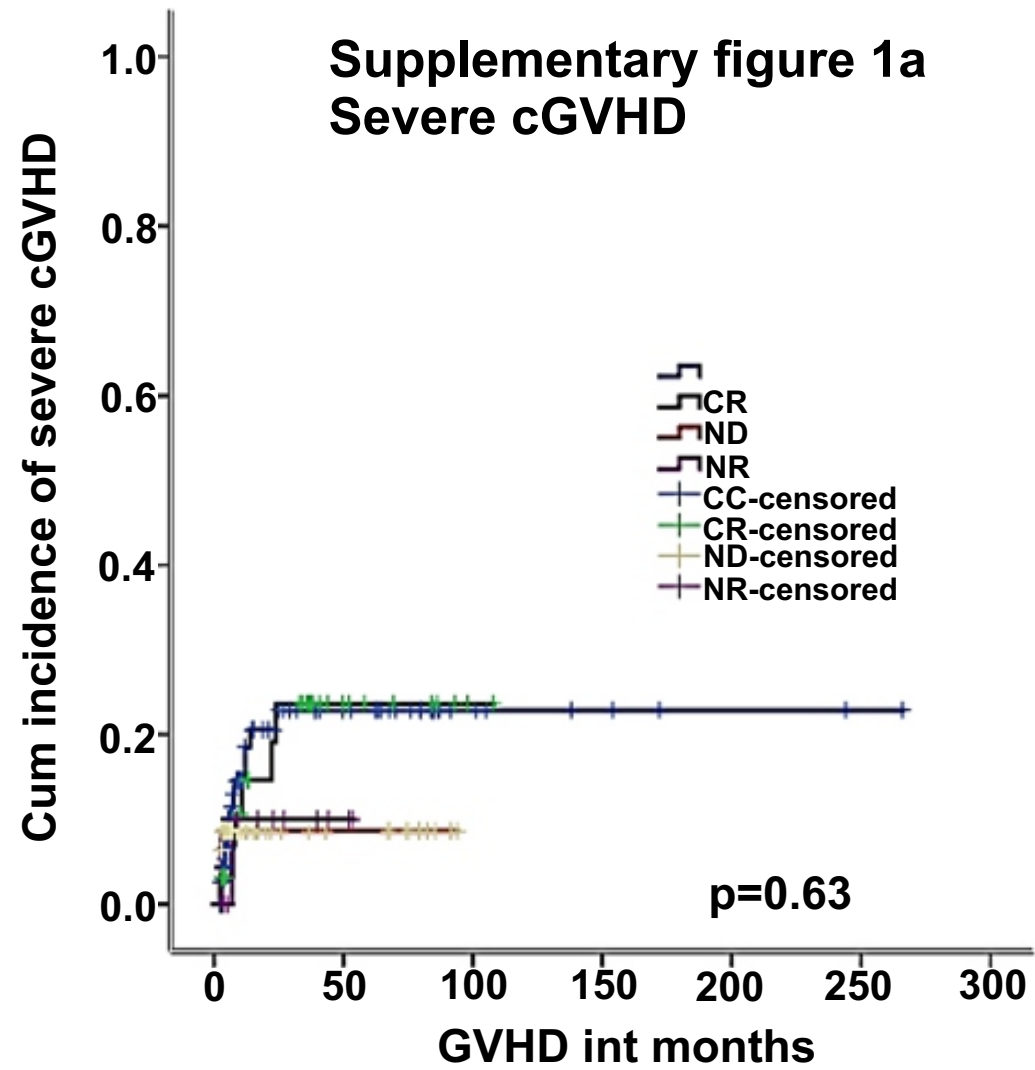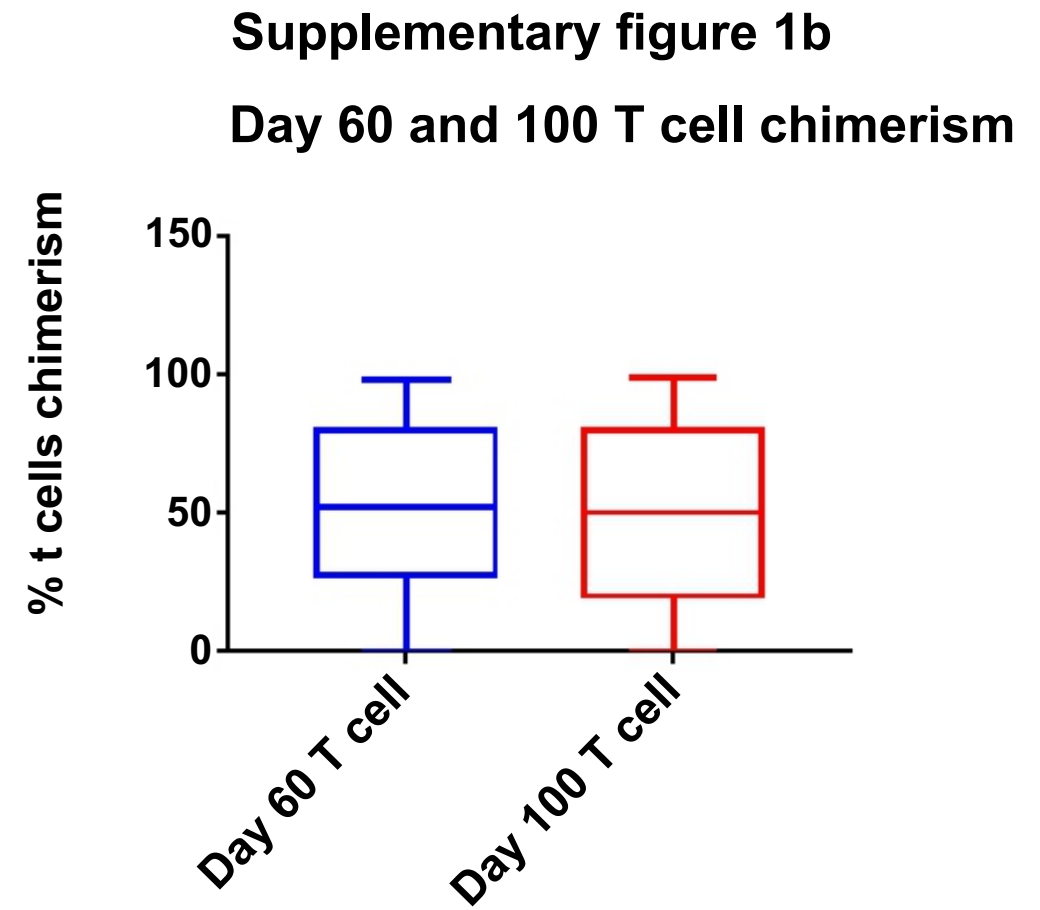

Supplement: Supplementary file 2 — Supplementary figure 1 [file 41408_2021_519_MOESM2_ESM.pdf]
